# Case Report: Severe persistent transaminitis during pregnancy: a complex differential diagnosis involving suspected toxoplasmosis, possible drug-induced liver injury, and intrahepatic cholestasis

**DOI:** 10.3389/fmed.2026.1965005

**Published:** 2026-09-29

**Authors:** Živilė Sabonytė-Balšaitienė, Jelena Volochovič

**Affiliations:** Clinic of Obstetrics and Gynecology, Institute of Clinical Medicine, Faculty of Medicine, Vilnius University, Vilnius, Lithuania

**Keywords:** case report, intrahepatic cholestasis of pregnancy, drug-induced liver injury, pregnancy, toxoplasmosis, transaminitis

## Abstract

**Introduction:**

Severe transaminitis during pregnancy represents a diagnostic challenge because multiple etiologies may coexist. We report a pregnant woman with suspected toxoplasmosis, intrahepatic cholestasis of pregnancy, and possible drug-induced liver injury who developed prolonged severe liver dysfunction despite extensive investigation and treatment.

**Case presentation:**

A 33-year-old primigravida was referred at 15 weeks' gestation because of positive Toxoplasma gondii serology and elevated liver enzymes (ALT/AST 460/150 U/L). Despite treatment with spiramycin, later changed to pyrimethamine-based therapy, and ursodeoxycholic acid, liver injury progressed (ALT/AST 1,363/466 U/L). Amniocentesis at 25 weeks showed negative T. gondii PCR. At 27 weeks, liver injury peaked (ALT/AST 1,734/728 U/L); anti-SS-A/Ro52 antibodies were detected, and bile acids subsequently increased to 82.4 μmol/L. Extensive investigations excluded viral hepatitis and did not support autoimmune hepatitis, while serial fetal assessment and amniotic fluid PCR showed no evidence of congenital toxoplasmosis. The clinical picture was considered most consistent with multifactorial liver injury involving possible drug-induced hepatotoxicity and intrahepatic cholestasis of pregnancy in the setting of suspected toxoplasmosis. Labor was induced at 38 weeks. A healthy male infant (3,140 g, Apgar scores 10/10) was delivered vaginally. Three days postpartum, ALT/AST decreased to 90/57 U/L.

**Conclusion:**

Severe transaminitis during pregnancy may have a multifactorial etiology, particularly when infectious, pregnancy-specific, medication-related, and immunological findings coexist. This case highlights the importance of careful differential diagnosis and multidisciplinary management when severe hepatocellular injury develops during pregnancy. Close maternal and fetal surveillance enabled the continuation of pregnancy to term, resulting in favorable maternal and neonatal outcomes.

## Introduction

Liver diseases during pregnancy represent a significant clinical challenge because they may adversely affect both maternal and fetal health. Although they complicate only approximately 3% of pregnancies, severe cases may result in substantial maternal and fetal morbidity and mortality (1). Liver diseases encountered during pregnancy can be broadly classified into three categories: pregnancy-specific disorders, liver diseases unrelated to pregnancy, and pre-existing chronic liver diseases (2). The physiological adaptations of pregnancy, including hormonal, immunological, and hepatobiliary changes, may influence liver function and further complicate the differential diagnosis of abnormal liver tests during gestation (3). The differential diagnosis of abnormal liver function tests during pregnancy is broad and includes pregnancy-specific disorders such as hyperemesis gravidarum, intrahepatic cholestasis of pregnancy, preeclampsia-related liver disease, and acute fatty liver of pregnancy (4). However, abnormal liver function tests may also result from viral infections, autoimmune liver disease, or drug-induced liver injury, often making the diagnostic evaluation challenging (5). Toxoplasmosis is one of the most common parasitic infections worldwide and remains an important concern during pregnancy because of the risk of congenital transmission. The risk of fetal infection increases with advancing gestational age, whereas disease severity tends to decrease (6). Most pregnant women are asymptomatic or present with mild, nonspecific symptoms (7). Although uncommon, hepatic manifestations of Toxoplasma gondii infection include hepatomegaly, granuloma formation, hepatitis, and hepatic necrosis (8). The coexistence of suspected toxoplasmosis and other potential causes of liver injury may present a significant diagnostic challenge in pregnant women with severe transaminitis. We report a pregnant woman with severe persistent transaminitis, suspected toxoplasmosis, intrahepatic cholestasis of pregnancy, positive autoimmune markers, and possible drug-induced liver injury. To our knowledge, reports describing the coexistence of suspected toxoplasmosis, severe hepatocellular injury, ICP, autoimmune markers, and possible drug-induced liver injury during a single pregnancy are extremely limited.

## Case description

A 33-year-old primigravida at 15 weeks of gestation was referred to an infectious diseases specialist after routine antenatal screening revealed positive Toxoplasma gondii IgG and IgM antibodies. Her medical history was notable for hypothyroidism treated with levothyroxine 50 μg daily and previously treated cervical dysplasia (ASC-H with high-risk HPV types 16, 51, and 66), for which cervical conization had been performed in 2017 and 2023. Epidemiological history revealed exposure to a domestic cat, contact with cat litter, consumption of raw meat before pregnancy, and ingestion of raw dandelion heads. The patient denied smoking and alcohol consumption.

Physical examination revealed no skin rash. The cardiorespiratory examination was normal. The liver edge was non-tender and splenomegaly was not detected. Mild left posterior cervical lymphadenopathy (1.0 × 1.5 cm) and bilateral axillary lymphadenopathy (1.0 × 1.0 cm) were present.

The clinical course is summarized in Table 1, while detailed laboratory findings, imaging studies, and treatment interventions are presented in Tables 2–4.

**Table 1:** Clinical timeline of the case.

Gestational age	Clinical findings and diagnostic assessment	Therapeutic intervention/outcome
15 weeks	Positive *Toxoplasma gondii* IgG and IgM; cervical and axillary lymphadenopathy; ALT 460 U/L, AST 150 U/L. IgG avidity 54.6%, suggesting infection before conception.	Spiramycin initiated for suspected maternal toxoplasmosis; hepatoprotective therapy started.
16–19 weeks	Persistent hepatocellular liver injury with progressive aminotransferase elevation.	Ursodeoxycholic acid added and dose-adjusted; IV fluid therapy subsequently initiated.
20 weeks	Persistent liver injury despite antiparasitic treatment.	Spiramycin discontinued; pyrimethamine with folinic acid initiated.
25 weeks	Amniotic fluid *T. gondii* PCR negative; fetal genetic testing normal. Persistent worsening of hepatocellular injury.	Pyrimethamine dose reduced because of concern for possible drug-induced hepatotoxicity.
26 weeks	Progressive liver injury; immune-mediated component considered.	Pyrimethamine discontinued; prednisolone 20 mg/day initiated.
27 weeks	Peak ALT 1,734 U/L and AST 728 U/L; strongly positive anti-SS-A/Ro52 antibodies with persistent lymphopenia.	Continued multidisciplinary diagnostic assessment and close maternal-fetal surveillance.
28 weeks	Increasing bile acids and persistent pruritus consistent with intrahepatic cholestasis of pregnancy.	Prednisolone tapered; ursodeoxycholic acid increased to 500 mg three times daily; cholestyramine added.
30–35 weeks	Aminotransferases fluctuated but gradually improved; bile acids remained elevated. Maternal and fetal conditions remained stable.	Intensive outpatient monitoring and IV fluid therapy 5 days/week.
35 weeks	Elevated fibrinogen and D-dimer without evidence of thrombosis.	Prophylactic bemiparin 3,500 IU/day initiated following hematological evaluation.
36 weeks	Re-admission for persistent ICP and liver dysfunction; substantial improvement in aminotransferases compared with peak values.	Multidisciplinary decision to continue pregnancy under close surveillance.
38 + 1 weeks	Stable maternal and fetal condition.	Labor induced; vaginal delivery of a healthy male infant, 3,140 g, Apgar scores 10/10.
Postpartum day 3	Marked biochemical improvement: ALT 90 U/L, AST 57 U/L, bile acids 5.8 μmol/L.	Uncomplicated postpartum course; discharged in good condition with outpatient hepatological follow-up.

ALT, alanine aminotransferase; AST, aspartate aminotransferase; ICP, intrahepatic cholestasis of pregnancy; IV, intravenous; PCR, polymerase chain reaction.

**Table 2:** Chronological changes in laboratory parameters during pregnancy and the postpartum period.

Main laboratory findings																							
Gestation (weeks)	15	16	17	18	19	20	21	23	24	25	26	27	28	29	30	31	32	34	35	36	37	38	PP day 4
Toxoplasma serology																							
Toxo-IgG (IU/mL)	1,776.5			1,666.6		1,145.6							549.9		332.1			177.0					
Toxo-IgM (s/co)	3.26					1.56							1.13										
IgG avidity test (%)	54.6					59.8																	
Liver enzymes																							
AST (GOT) (U/L)	150	200 →107	174	181	157	111	93	62	406	466 →434	414	728	573 →542	452	576 → 663	442	460 →249	143	137	150	104	90	57
ALT (GPT) (U/L)	460	572 →404	589	699	613	433	329	177	1,088	1,363 →1,193	1,043	1,734	1,489 →1,455	1,038	1,123 →1,085	782	799 →420	245	239	257	181	159	90
Bile acids (µmol/L)		10.8 →3.3	4.0	6.7	14.2	7.3	6.0	21.3	10.2	10	8.3	23.6	12.8 →22.6	31.8	44.2 →48.8	82.4	54.9 →21.9	15.1		16.6	22.2	23.5	5.8
Lymphopenia (×10⁹/L, %)	1.27, 17.0	0.77, 13.5		0.84, 16.0						0.87, 11.8	0.82, 11.7		0.74, 7.3	0.96, 14.7						0.97, 18.0	0.94, 20.3		
Fibrinogen (g/L)		4.42		4.94									6.53 →6.72	6.72		7.29	6.43 →7.52	8.6	9.05	7.74	7.44	7.10	5.72
Bilirubin (µmol/L)		5.3 →7.9	4.8	6.3	6.2	6.2	5.6	3.8	9.3	9.3	8.1	6.5	8.3 →8.8	9.5	10.8 →10.9	10.0	13.3 →8.8	10.0		8.7	13.1	11.7	7.8
Immunological testing																							
AMA M2 (IU/mL)		Negative, 1.0																					
Antinuclear antibodies (ANA)		Positive, titer 1:160																					
Complement components (g/L)		C3 1.4 C4 0.5									C3 1.73 C4 0.24												
Autoantibodies												Anti-SS-A (+++), anti-Ro52 (+++); remaining ENA profile, anti-dsDNA negative.											
Lymphocyte immunophenotyping												Lymphocytes 583/mm³; CD4/CD8 ratio 0.8.											

Values are presented as peak values or ranges when repeated measurements were performed during the same assessment period.

**Table 3:** Chronological changes in fetal and maternal ultrasound findings during pregnancy.

Gestation (weeks + days)	15 + 3	17 + 3	19 + 5	20 + 6	22 + 2	23 + 2	24 + 4	25	26 + 4	27 + 3	28 + 3	29 + 2	30 + 3	31 + 3	32 + 2	33 + 6	34 + 2	35 + 6	37 + 4
Fetal ultrasound	Normal anatomy; EFW 123 ± 18 g.	Detailed anomaly scan normal; male fetus.	EFW 312 ± 46 g (72nd percentile).	Normal growth, EFW 389 g.	Normal growth, EFW 630 g.	Mild prominence of left lateral ventricular posterior horn (8.5 mm).	Normal fetal growth and wellbeing.		Ventricular measurements normalized (5.0 and 4.5 mm).	Normal fetal growth and wellbeing.	Right ureteral dilatation 7.6 mm.	Persistent ureteral dilatation (8 mm); mild bowel dilatation	LGA fetus; AC >97.7th percentile. Stable ureteral dilatation (6.7 mm).	Normal fetal wellbeing.	Normal fetal wellbeing, EFW 2,212 ± 323 g (77th percentile).	Normal fetal wellbeing.	Persistent ureteral dilatation (10 mm).	Normal fetal wellbeing, cervical length 2.43 cm with a closed internal os.	EFW 3,025 ± 442 g; cephalic presentation.
Maternal ultrasound	Normal liver morphology and vascular flow. Mild bilateral renal pelvic dilatation and splenomegaly.							Normal liver morphology; no biliary dilatation or vascular thrombosis. Probable 12-mm hepatic hemangioma.			Normal liver morphology; F0 fibrosis, steatosis <3%.								

All serial ultrasound examinations demonstrated normal fetal growth, preserved fetoplacental circulation, and no sonographic signs of congenital toxoplasmosis unless otherwise specified.

**Table 4:** Chronological changes in treatment during pregnancy.

Treatment		
	Medications	Main changes
Gestation (weeks)		
15	spiramycin 1.5 million IU twice daily hepatoprotective therapy: silymarin 140 mg twice daily, essential phospholipids 600 mg once daily, probiotics (Alflorex once daily).	First hospitalization; spiramycin initiated
16	spiramycin 1.5 million IU twice daily hepatoprotective therapy: silymarin 140 mg twice daily, essential phospholipids 600 mg once daily, probiotics (Alflorex once daily). ursodeoxycholic acid 500 mg twice daily	Ursodeoxycholic acid added
17–18	spiramycin 1.5 million IU twice daily hepatoprotective therapy: silymarin 140 mg three times daily, essential phospholipids 600 mg once daily, probiotics (Alflorex once daily). ursodeoxycholic acid 250 mg in the morning and 500 mg in the evening	Ursodeoxycholic acid dose increased Silymarin dose increased
19	spiramycin 1.5 million IU twice daily hepatoprotective therapy: silymarin 140 mg three times daily, essential phospholipids 600 mg once daily, probiotics (Alflorex once daily). ursodeoxycholic acid 250 mg in the morning and 500 mg in the evening IV Ringer's solution 1,000 mL + 0.9% sodium chloride 500 mL.	Weekly IV infusion therapy initiated
20	Pyrimethamine 25 mg twice daily for 1 day, followed by 25 mg once daily with folinic acid 7.5 mg once daily. hepatoprotective therapy: silymarin 140 mg three times daily, essential phospholipids 600 mg once daily, probiotics (Alflorex once daily). ursodeoxycholic acid 250 mg in the morning and 500 mg in the evening IV Ringer's solution 1,000 mL + 0.9% sodium chloride 500 mL.	Spiramycin discontinued Pyrimethamine initiated
25	Pyrimethamine 25 mg twice weekly (Tuesdays and Fridays); folinic acid 7.5 mg daily on the remaining 5 days of the week. hepatoprotective therapy: silymarin 140 mg three times daily, essential phospholipids 600 mg once daily, probiotics (Alflorex once daily). ursodeoxycholic acid 250 mg in the morning and 500 mg in the evening IV Ringer's solution 1,000 mL + 0.9% sodium chloride 500 mL.	Second hospitalization; pyrimethamine reduced to twice weekly
26	Prednisolone 20 mg once daily hepatoprotective therapy: silymarin 140 mg three times daily, essential phospholipids 600 mg once daily, ursodeoxycholic acid 250 mg in the morning and 500 mg in the evening	• Pyrimethamine and folinic acid discontinued Prednisolone initiated
28	Prednisolone tapering regimen hepatoprotective therapy: silymarin 140 mg three times daily, essential phospholipids 600 mg once daily, Cholestyramine 4 g up to 4 times per day ursodeoxycholic acid 500 mg three times per day	Third hospitalization; treatment modified (prednisolone tapered, ursodeoxycholic acid increased, cholestyramine added)
30	hepatoprotective therapy: silymarin 140 mg three times daily, essential phospholipids 600 mg once daily, Cholestyramine 4 g up to 4 times per day ursodeoxycholic acid 500 mg three times per day IV Ringer's solution 1,000 mL + 0.9% sodium chloride 500 mL.	IV infusion therapy intensified to 5 days/week
30–35	Maintenance therapy unchanged; IV infusions continued 5 days/week; lactulose, Microlax and omeprazole PRN.	
35	Bemiparin 3,500 IU SC once daily	Bemiparin initiated

Significant hepatocellular liver injury was observed with ALT 460 U/L and AST 150 U/L, while HBsAg, HCV antibodies, and HIV serology were negative. Mild lymphopenia was also detected. Although the avidity results suggested infection acquired before conception, persistently positive IgM antibodies and uncertainty regarding the timing of infection prompted initiation of antiparasitic therapy.

### Diagnostic assessment

Because of progressive hepatocellular liver injury, the patient underwent extensive multidisciplinary evaluation involving obstetricians, infectious diseases specialists, gastroenterologists, clinical pharmacologists, clinical geneticists, an allergist-immunologist, a rheumatologist, a hematologist, and a neonatologist. Abdominal ultrasonography and hepatic vascular Doppler examination initially demonstrated normal liver morphology and vascular flow. Subsequent examinations revealed no significant structural or vascular liver pathology, except for a probable 12-mm hepatic hemangioma.

Extensive infectious investigations excluded hepatitis B, C, and E, CMV, EBV, HSV, VZV, adenovirus, and parvovirus B19 infection. Serial Toxoplasma gondii serology demonstrated persistently positive IgM antibodies and progressively decreasing IgG concentrations. The initial IgG avidity index was 54.6%, suggesting infection acquired before conception. At 25 weeks of gestation, diagnostic amniocentesis demonstrated negative T. gondii PCR, while fetal genetic testing revealed no chromosomal abnormalities. Serial fetal ultrasonography showed normal growth and well-being without sonographic evidence of congenital toxoplasmosis.

Metabolic and vascular causes of liver injury were not identified. Preeclampsia-related liver disease was considered unlikely because angiogenic markers were normal (PlGF 470.5 ng/L; sFlt-1/PlGF ratio 4.62). Autoimmune testing demonstrated positive ANA (1:160), borderline elevated total IgG (16.59 g/L), and negative AMA-M2; however, the available clinical and laboratory findings were insufficient to establish autoimmune hepatitis. Subsequent immunological evaluation revealed strongly positive anti-SS-A and anti-Ro52 antibodies, persistent lymphopenia, and a reduced CD4/CD8 ratio, whereas the remaining ENA profile and anti-dsDNA antibodies were negative.

Despite extensive investigation, no single etiology could explain the severity and persistence of the hepatocellular injury. The differential diagnosis therefore included possible T. gondii-associated liver involvement, possible drug-induced liver injury related to antiparasitic therapy, an underlying immune-mediated process, and subsequently developing intrahepatic cholestasis of pregnancy.

### Therapeutic intervention

Treatment with spiramycin was initiated at 15 weeks of gestation because of suspected maternal toxoplasmosis, together with supportive hepatoprotective therapy. Exact medication doses, dosing frequencies, and treatment periods are summarized in Table 4. Ursodeoxycholic acid was added at 16 weeks because of persistent liver enzyme elevation and was subsequently dose-adjusted according to the biochemical course. Despite treatment, aminotransferase levels continued to increase, and supportive intravenous fluid therapy was introduced.

At 20 weeks of gestation, spiramycin was discontinued and treatment was switched to pyrimethamine with folinic acid supplementation following consultation with a clinical pharmacologist. Because of persistent and subsequently worsening hepatocellular injury and concern regarding possible drug-induced hepatotoxicity, the pyrimethamine dose was reduced at 25 weeks and the treatment was discontinued at 26 weeks.

Given the persistent severe hepatocellular injury of unclear etiology together with positive ANA, strongly positive anti-SSA/Ro52 antibodies, persistent lymphopenia, and a reduced CD4/CD8 ratio, an immune-mediated component was considered possible, and prednisolone 20 mg daily was initiated at 26 weeks. However, no significant biochemical improvement was observed, and corticosteroid therapy was gradually tapered and discontinued. With the subsequent development of intrahepatic cholestasis of pregnancy, treatment was intensified at 28 weeks with ursodeoxycholic acid 500 mg three times daily and cholestyramine 4 g up to four times daily, while hepatoprotective therapy was continued. Intravenous fluid therapy was subsequently intensified to five days per week because of fluctuating liver enzyme levels and increasing bile acid concentrations.

At 35 weeks of gestation, prophylactic bemiparin sodium 3,500 IU, administered subcutaneously once daily, was initiated after a marked increase in fibrinogen to 9 g/L. Subsequent hematological evaluation showed D-dimer levels of 1,000–1,200, a normal platelet count, and decreasing fibrinogen levels. The hematologist considered the fibrinogen elevation to be consistent with physiological changes of the third trimester and recommended continued clinical surveillance for thrombosis.

The temporal relationship between laboratory findings, treatment, and key clinical events is summarized in Figure 1.

**Figure 1:**
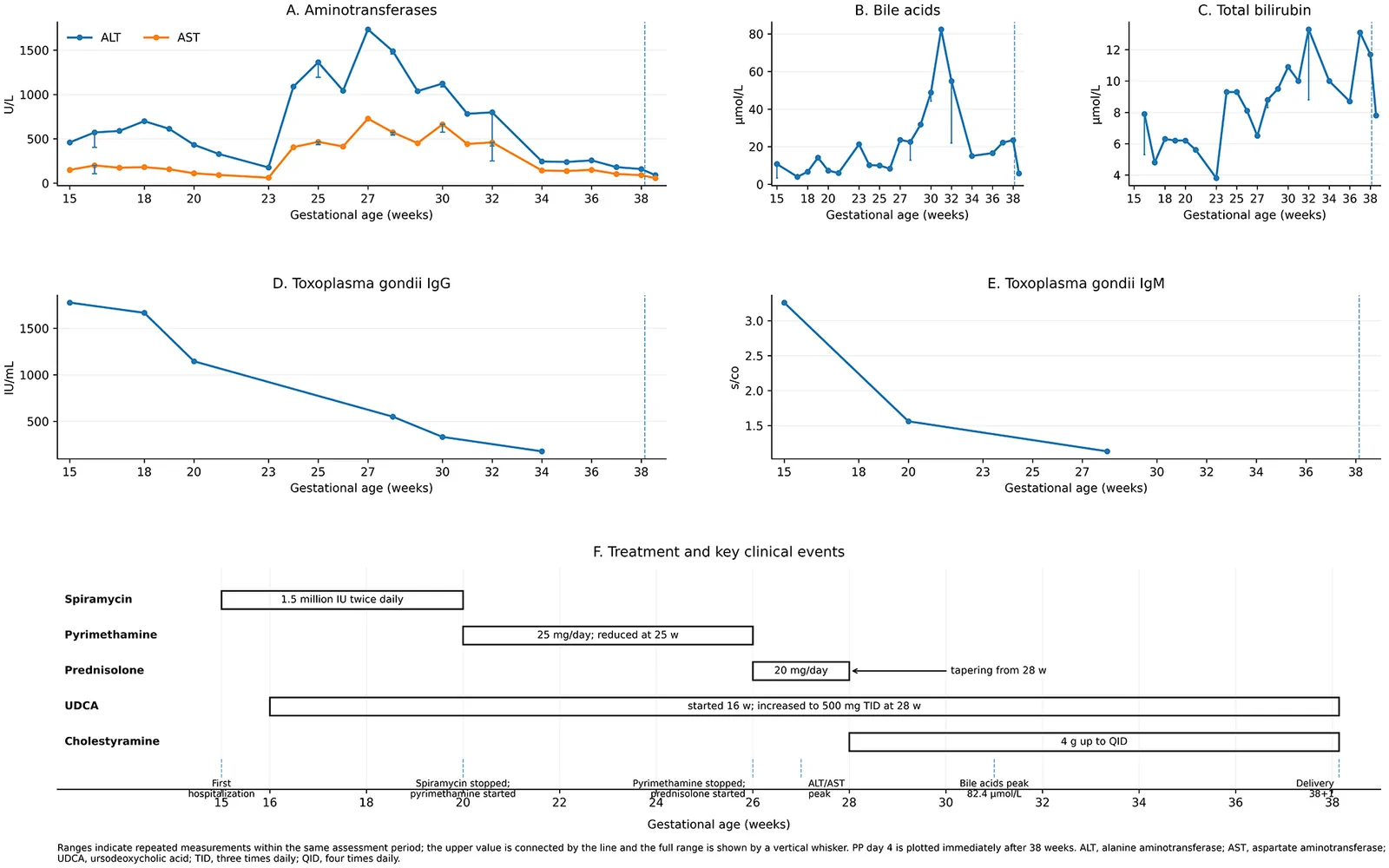
Clinical course during pregnancy. (**A**) ALT and AST concentrations; (**B**) bile acid concentrations; (**C**) total bilirubin concentrations; (**D**) Toxoplasma gondii IgG; (**E**) Toxoplasma gondii IgM; and (**F**) treatment and key clinical events. ALT, alanine aminotransferase; AST, aspartate aminotransferase; UDCA, ursodeoxycholic acid.

### Follow-up and outcomes

From 28 weeks of gestation onward, the patient remained under close multidisciplinary follow-up in the obstetric day care unit, with serial assessment of liver enzymes, bile acid concentrations, coagulation parameters, and maternal symptoms. Maternal liver function gradually improved after discontinuation of antiparasitic and corticosteroid therapy, although aminotransferase levels remained fluctuating and bile acid concentrations continued to increase, reaching a peak of 82.4 μmol/L. Pruritus worsened transiently but subsequently resolved with intensified treatment. Hepatic synthetic function remained preserved throughout the clinical course, with INR values within the normal range. Serum albumin was 32 g/L at 25 weeks, and total bilirubin remained within the normal range. GGT remained largely unremarkable, whereas ALP increased during late pregnancy. Direct bilirubin measurements were not available.

Close fetal surveillance was maintained throughout the third trimester, including serial ultrasonography, Doppler assessment, and non-stress testing. Fetal growth and well-being remained reassuring, with no sonographic evidence of congenital toxoplasmosis or fetal cardiac involvement related to maternal anti-SS-A/Ro52 antibodies. Mild transient dilatation of the fetal right ureter and large bowel was observed during follow-up without evidence of progressive fetal pathology. Serial *Toxoplasma gondii* serology showed a progressive decline in maternal IgG titers, and re-initiation of antiparasitic therapy was not considered indicated.

At 36 weeks of gestation, the patient was re-admitted for evaluation of persistent intrahepatic cholestasis of pregnancy and liver dysfunction of uncertain etiology. Maternal liver biochemistry had substantially improved compared with peak values, and maternal and fetal conditions remained stable. Following multidisciplinary assessment, pregnancy was continued to 38 weeks, when induction of labor was planned because of persistent intrahepatic cholestasis and elevated liver enzymes.

At 38 + 1 weeks of gestation, following induction with oral misoprostol and oxytocin augmentation, the patient delivered a healthy male infant vaginally. Birth weight was 3,140 g, length 53 cm, and Apgar scores were 10 and 10 at 1 and 5 min, respectively. No clinical signs suggestive of congenital toxoplasmosis were observed. The postpartum course was uncomplicated. Three days after delivery, liver biochemistry showed marked improvement, with ALT decreasing to 90 U/L, AST to 57 U/L, and bile acids to 5.8 *μ*mol/L. The patient was discharged in good condition with recommendations for outpatient hepatological follow-up.

## Discussion

Toxoplasmosis and intrahepatic cholestasis of pregnancy are well-recognized conditions that may complicate pregnancy. However, the coexistence of severe and persistent liver injury in this setting presents a considerable diagnostic challenge. This case highlights the complexity of evaluating severe hepatocellular injury in the setting of suspected maternal toxoplasmosis and subsequent intrahepatic cholestasis of pregnancy, while also raising the possibility of drug-induced liver injury associated with antiparasitic therapy. We believe this report serves as an important reminder that marked transaminitis during pregnancy may have a multifactorial etiology and should not be attributed solely to more common pregnancy-specific liver disorders such as preeclampsia, HELLP syndrome, or acute fatty liver of pregnancy.

Toxoplasmosis is a zoonotic infection caused by the obligate intracellular protozoan parasite Toxoplasma gondii. It is one of the most common chronic parasitic infections worldwide, affecting approximately one-third of the global population. The prevalence of toxoplasmosis varies considerably across geographic regions and is influenced by climatic conditions, dietary habits, and environmental exposures. Among women of reproductive age in industrialized countries, seroprevalence rates range from 10% to 50%. Higher rates, reaching up to 80%, have been reported in certain tropical regions where exposure to contaminated soil, undercooked meat, or untreated water is common (9, 10). Primary maternal infection during pregnancy is of particular concern because of the risk of transplacental transmission and potentially severe fetal consequences, including neurological and ophthalmological complications (11). Establishing whether primary maternal infection occurred during pregnancy requires careful interpretation of Toxoplasma-specific IgG and IgM antibodies together with IgG avidity and serial serological testing. Persistent IgM positivity alone does not necessarily indicate recent primary infection, whereas higher IgG avidity, particularly when detected early in pregnancy, may support infection acquired before conception. Therefore, serological findings should be interpreted together with their temporal evolution and, when indicated, fetal assessment and amniotic-fluid PCR. Acute toxoplasmosis is asymptomatic in more than 80% of immunocompetent individuals. When symptoms occur, they are typically mild and non-specific, including fever, headache, myalgia, fatigue, pharyngitis, and lymphadenopathy. Cervical lymphadenopathy is the most common clinical manifestation and may persist for several weeks. Less frequently, hepatosplenomegaly and skin rash may be observed. Although hepatic involvement is uncommon, elevated liver enzymes and, rarely, clinically significant hepatitis have been described in patients with acute toxoplasmosis. Experimental studies further support a direct role of Toxoplasma gondii in hepatic injury. In a murine model of acute toxoplasmosis, T. gondii macrophage migration inhibitory factor (TgMIF) promoted hepatocyte pyroptosis and recruitment of proinflammatory macrophages, resulting in severe inflammatory liver damage. These findings suggest that parasite-induced immune activation may contribute to hepatocellular injury during acute toxoplasmosis (12). Consequently, toxoplasmosis should be considered among the differential diagnoses of unexplained liver injury, particularly when accompanied by compatible clinical and serological findings (9). At presentation, toxoplasmosis was considered in the differential diagnosis because several clinical and serological findings raised suspicion of maternal T. gondii infection. The patient presented with fatigue, pruritus, cervical and axillary lymphadenopathy, markedly elevated Toxoplasma IgG titres, persistent Toxoplasma IgM positivity, and elevated liver enzymes. However, the timing of maternal infection could not be established. The initial Toxoplasma IgG avidity index was elevated (54.6%), suggesting that infection may have been acquired before conception rather than during pregnancy. Furthermore, serial serological testing demonstrated a progressive decline in Toxoplasma IgG titres. Repeated fetal ultrasonographic examinations remained unremarkable throughout pregnancy, showing no sonographic signs of congenital toxoplasmosis, and amniotic-fluid polymerase chain reaction testing for Toxoplasma gondii was negative. Importantly, liver enzyme levels continued to increase despite antiparasitic treatment, reaching peak values of ALT 1,734 U/L and AST 728 U/L. Taken together, these findings indicate that toxoplasmosis was an important initial diagnostic consideration; however, its contribution to the severe and persistent hepatocellular injury remained uncertain, and the available evidence did not establish acute primary maternal infection acquired during pregnancy or fetal infection.

Drug-induced liver injury (DILI) emerged as an important alternative diagnostic consideration in our patient. Spiramycin has been used clinically for decades and is generally considered well tolerated, with gastrointestinal adverse effects and mild cutaneous reactions being the most frequently reported side effects. Unlike several other macrolides, convincing evidence linking spiramycin monotherapy to clinically significant hepatitis remains limited (13). Hussein et al. described four patients who developed moderate-to-severe liver injury after exposure to a spiramycin-metronidazole combination, including one patient who required liver transplantation because of subacute massive hepatic necrosis (14). Because metronidazole was co-administered in these cases, causality could not be attributed specifically to spiramycin, and this report therefore provides only limited evidence for spiramycin-associated hepatotoxicity. Evidence regarding pyrimethamine-associated hepatotoxicity remains limited. However, pyrimethamine-containing regimens have been linked to rare cases of clinically apparent liver injury, including severe hepatocellular damage and acute liver failure (15). It remains unclear whether pyrimethamine alone can cause liver injury, as most reported cases occurred during combination therapy with sulfonamides. In an analysis of pediatric hepatic adverse events reported to the U.S. Food and Drug Administration Adverse Event Reporting System, pyrimethamine was identified among several newly detected drugs potentially associated with liver injury. Although causality could not be established, these findings indicate a possible association between pyrimethamine-containing therapy and liver injury that warrants further investigation (16). In our patient, aminotransferase levels continued to rise despite improving toxoplasmosis serological markers, absence of fetal infection, and lack of sonographic evidence of congenital toxoplasmosis. Furthermore, the most pronounced biochemical deterioration occurred during prolonged antiparasitic therapy, while serological markers of toxoplasmosis were gradually improving. However, because liver biopsy was not performed and multiple potential etiologies coexisted, a definitive diagnosis of DILI could not be established. The temporal relationship between antiparasitic therapy and progressive hepatocellular injury raised the possibility of DILI; however, its contribution to the overall clinical course remained uncertain. Given the presence of liver injury before antiparasitic treatment, multiple sequential drug exposures, and several competing etiologies, a retrospective RUCAM assessment was considered unlikely to provide a reliable causality estimate in this case.

The interpretation of this case was further complicated by strongly positive anti-SS-A and anti-Ro52 antibodies, accompanied by persistent lymphopenia. Anti-Ro52 antibodies have been described in a broad spectrum of autoimmune disorders and are generally regarded as markers of immune activation rather than disease-specific biomarkers. In pregnancy, anti-SSA/Ro antibodies are clinically relevant because of their association with neonatal lupus and congenital heart block (17, 18). Serial fetal ultrasonographic surveillance remained unremarkable throughout pregnancy, and no evidence of fetal congenital abnormalities or diseases was detected. Although these findings raised the possibility of an underlying immune-mediated process, our patient lacked additional serological and clinical features required to establish a diagnosis of autoimmune hepatitis or another defined systemic autoimmune disease. Therefore, while an autoimmune contribution cannot be completely excluded, the available evidence was insufficient to support a primary autoimmune etiology of the observed liver injury.

Intrahepatic cholestasis of pregnancy (ICP) is one of the most common pregnancy-specific liver disorders and typically manifests during the second or third trimester of pregnancy. A recent systematic review and meta-analysis including more than 42 million pregnancies estimated the global incidence of ICP at 2.9%, although substantial geographical variation exists, with the highest incidence reported in Asia and lower rates observed in developed countries (19). Clinically, ICP is characterized by pruritus and elevated serum bile acid concentrations and is associated with an increased risk of adverse perinatal outcomes (20). These adverse outcomes include stillbirth, spontaneous preterm birth, meconium-stained amniotic fluid, and neonatal intensive care unit admission (21).

The subsequent clinical course strongly suggested an additional contribution of ICP. In our patient, pruritus persisted throughout pregnancy and was accompanied by a progressive rise in serum bile acid levels, reaching a peak concentration of 82.4 μmol/L despite treatment with ursodeoxycholic acid. These findings fulfilled the diagnostic criteria for ICP and supported its presence as a significant component of the disease process. However, although elevated aminotransferase levels are frequently observed in ICP, liver enzyme elevations are typically mild to moderate and are not considered the defining biochemical feature of the disease (21). In our patient, peak ALT and AST concentrations reached 1,734 U/L and 728 U/L, respectively, far exceeding the elevations usually reported in ICP. Although rare cases of severe transaminasemia associated with ICP have been described in the literature (22), such presentations remain uncommon. Therefore, ICP alone is unlikely to account for the marked and persistent hepatocellular injury observed in this case.

The subsequent improvement in liver biochemistry and bile acid concentrations following delivery further supports the contribution of pregnancy-related cholestasis to the later stages of the clinical course. This observation is consistent with the natural history of ICP, in which pruritus typically resolves rapidly after birth, whereas biochemical abnormalities may persist longer before returning to baseline (20).

A major strength of this case is the extensive multidisciplinary diagnostic evaluation, longitudinal biochemical monitoring, serial assessment of *Toxoplasma gondii* serology, and close maternal-fetal surveillance throughout pregnancy. However, several limitations should be acknowledged. The precise etiology of liver injury could not be definitively established because multiple potentially contributing factors coexisted. Liver biopsy was not performed because it was not expected to alter clinical management, limiting histopathological assessment of possible drug-induced or immune-mediated liver injury. Complete autoimmune hepatitis-specific serological testing, including SMA, anti-LKM1, and anti-SLA/LP antibodies, was not available, further limiting definitive exclusion of an autoimmune etiology. Furthermore, the timing of maternal T. gondii infection could not be established with certainty despite serial serological testing.

Taken together, this case represents severe hepatocellular injury of uncertain and potentially multifactorial etiology during pregnancy. Serologically suspected maternal T. gondii infection was an important consideration during the initial clinical presentation; however, its contribution to the hepatocellular injury remained uncertain. Possible drug-induced liver injury associated with antiparasitic therapy was also considered, although causality could not be established. The subsequent development of intrahepatic cholestasis of pregnancy further complicated the clinical course and likely contributed to the later biochemical abnormalities. This case highlights the importance of maintaining a broad differential diagnosis when evaluating severe aminotransferase elevation during pregnancy and illustrates how infectious, drug-related, autoimmune, and pregnancy-specific factors may coexist in a single patient.

## Patient perspective

A patient perspective was not obtained for inclusion in this case report.

## Data Availability

The original contributions presented in the study are included in the article/Supplementary Material, further inquiries can be directed to the corresponding author.
